# Oxytocin, Weight Loss and Ketosis in Response to a Very-Low-Calorie Ketogenic Diet: An Exploratory Study

**DOI:** 10.3390/nu18030485

**Published:** 2026-02-01

**Authors:** Elena Gangitano, Rebecca Rossetti, Rossella Tozzi, Paola Nevi, Davide Masi, Sabrina Basciani, Orietta Gandini, Mikiko Watanabe, Mariaignazia Curreli, Lucio Gnessi, Stefania Mariani, Carla Lubrano

**Affiliations:** 1 Department of Experimental Medicine, Sapienza University of Rome, 00161 Rome, Italy; 2 Departmental Faculty of Medicine, Unicamillus-Saint Camillus International University of Health Sciences, 00131 Rome, Italy; 3 Department of Molecular Medicine, Sapienza University of Rome, 00161 Rome, Italy; 4 Unit of Endocrinology, Fondazione Policlinico Universitario Campus Bio-Medico, 00128 Rome, Italy

**Keywords:** oxytocin, obesity, excess weight, overweight, weight loss, ketogenic diet, ketosis, VLCKD

## Abstract

**Background/Objectives:** Obesity is a chronic relapsing disorder associated with many comorbidities. Some evidence suggests that oxytocin (OT) has an anorexigenic effect, but its levels are often increased in obesity. This study investigates the effects of weight loss induced by a very-low-calorie ketogenic diet (VLCKD) on oxytocin levels. **Methods:** A total of 47 subjects with overweight or obesity, 28 females (60%) and 19 males, with a mean age of 55.5 ± 7.3 years and mean BMI 35.9 ± 4.4 kg/m^2^, underwent VLCKD for 45 days. We assessed anthropometric parameters, metabolic profile, body composition and OT levels at baseline (t0) and at the end of the diet (t1). **Results:** After weight loss, plasma OT levels significantly dropped. Baseline OT correlated with BMI, fat mass and trunk fat. A linear relationship was observed between Delta OT levels and Delta BMI. Baseline OT was an independent predictor of weight loss and directly correlated with blood ketone levels at the end of the study. An optimal serum OT cut-off that predicts ketosis occurrence was identified. **Conclusions:** Weight loss obtained with a VLCKD reduces OT levels in patients with excess weight. Baseline OT predicts weight loss and correlates with ketone body levels during a VLCKD.

## 1. Introduction

Obesity is a chronic disease that has reached pandemic proportions, and its prevalence is expected to increase in the near future [1]. It is characterized by a complex and multifaceted pathophysiology and is frequently associated with other metabolic diseases, such as type 2 diabetes and hepatic steatosis [2]. Obesity negatively affects the cardiovascular system and immune response, and is associated with increased susceptibility to certain types of cancer, infectious diseases, and psychological issues [3,4,5,6,7].

Oxytocin (OT) is a neuropeptide produced in the paraventricular, supraoptic, and accessory nuclei of the hypothalamus, and acts as a neurohormone, neuromodulator and neurotransmitter. Its actions are tissue-specific and influenced by complex neural and endocrine interactions. OT’s main effect is promoting uterus contraction during delivery and milk let-down during lactation. However, oxytocin is also related to social behaviour and metabolic homeostasis [8,9]. OT enhances glucose uptake and lipid oxidation in adipose tissue and skeletal muscle [10], but interestingly, favourable glucose homeostasis effects appear to be decreased with increased adiposity [9]. Moreover, OT regulates energy balance by influencing appetite; in fact, OT has an anorexigenic effect and reduces hedonic food intake [9]. OT levels have been reported to be high in obesity and in metabolic syndrome, even if not all studies are concordant [9,11,12]. Oppositely, plasma OT was reduced in underweight women, with a directly proportional relationship with BMI reported [13,14,15]. Surprisingly, intranasal administration of OT induces weight loss and reduces caloric intake and hedonic food intake [9], and these effects are more pronounced in individuals with obesity than in normal-weight subjects or overweight patients [16,17]. These elements suggest altered OT signalling in subjects with obesity. The hypothesis that a dysfunction occurring in the OT/OT-R system could promote weight gain has been advanced [16,18,19]. Nevertheless, the precise physiological role of OT in regulating body weight, metabolic health, and psychological aspects in individuals with obesity remains unclear.

A very-low-calorie ketogenic diet (VLCKD) is a kind of KD characterized by a very low carbohydrate and caloric intake, and in selected patients is a valid option for a significant weight loss [20].

We wanted to investigate plasma OT modifications in individuals affected by weight excess at baseline and after weight loss induced by a VLCKD. Considering the efficacy and speed of a VLCKD in reducing body weight, we decided to study changes in OT values in a pool of patients at baseline and following a 45-day period of VLCKD.

## 2. Materials and Methods

This was a single-centre prospective observational study that enrolled patients with excess weight prescribed a VLCKD, attending the CASCO Centre (High-Specialization Centre for the Care of Obesity), Department of Experimental Medicine, Section of Medical Pathophysiology, Food Science and Endocrinology, Umberto I Polyclinic, Sapienza University of Rome, Italy. Patients were enrolled from January 2021 to December 2023.

Inclusion criteria were as follows: BMI between 25 and 45 kg/m^2^, stable body weight in the previous 3 months, and age between 35 and 75 years. Exclusion criteria were: severe organ failure, recent myocardial infarction, psychiatric diseases possibly hindering compliance, type 1 diabetes and beta cell failure, use of SGLT-2 inhibitors, lack of informed consent, pregnancy or breastfeeding and previous bariatric surgery.

Patients were evaluated at baseline (t0) and after 45 days of a VLCKD (t1). All patients underwent a comprehensive clinical evaluation and biochemical routine assessment. Every two weeks, patients attended follow-up visits to monitor their adherence to the diet and assess any adverse effects. Adverse events were monitored throughout the study.

### 2.1. Anthropometric Parameters

The anthropometric measurements included weight (kg), height (cm) and waist circumference (WC—cm); vital signs collected included systolic blood pressure (SBP—mmHg), diastolic blood pressure (DBP—mmHg) and heart rate (HR—bpm).

Body weight and height were measured between 8:00 and 10:00 AM in fasting and empty-bladder subjects, with light clothing and without shoes. A calibrated balance beam (200 kg ± 0.1 kg) and a stadiometer (200 cm ± 0.1 cm) were used for recording weight and height, respectively (Seca GmbH & Co, Hamburg, Germany). WC was measured using a flexible, inelastic tape measure positioned at the anterior superior iliac spine. The body mass index (BMI) was calculated as weight in kilograms divided by the square of the height in meters. Blood pressure was measured by Gamma XXL LF-S 2-tube rail aneroid sphygmomanometer (HEINE Optotechnik, Herrsching, Germany) using the dedicated cuff for obesity.

### 2.2. Blood and Urine Chemistry

Peripheral blood samples were collected from participants at 8.00 AM after an overnight fast of at least 8 h in EDTA tubes. The biochemical assessment included: fasting plasma glucose (FPG), fasting serum insulin, glycosylated hemoglobin A1C (HbA1c), lipid evaluation including triglycerides, total cholesterol and HDL cholesterol; low-density lipoprotein cholesterol (cLDL) was calculated via the Friedwald formula. The evaluation of the inflammatory state involved measuring C-reactive protein (CRP) levels (µg/L) using the immunoturbidimetric method.

Serum oxytocin concentration was performed on plasma using a commercial ELISA kit (ABCAM, cod. Ab133050, Cambridge, UK). The sample was properly prepared by adding 500 KIU of Aprotinin (ABCAM cod. ab146286, Cambridge, UK) per 10 mL of whole blood. The aprotinin was added within the first 5 min of collection, and the sample was centrifuged for 14 min at 1400 rpm immediately after the addition of the aprotinin. Once the sample was centrifuged, the plasma was stored at −80 °C until further analysis.

Plasma ketone bodies were assessed by β-OH-butyrate assay (Colorimetric Assay Kit Cayman, Ann Arbor, MI, USA) according to the manufacturer’s instructions. Plasma samples were collected after an overnight fast, centrifuged to separate cellular components, and stored at −80 °C until analysis. Plasma ketone bodies were assessed at baseline and at the end of the dietary intervention.

### 2.3. Dual-Energy X-Ray Absorptiometry and Body Composition

Body composition was obtained by dual energy X-ray absorptiometry (DXA) technique using Hologic Inc., Bedford, MA, USA, QDR 4500W Discovery A machinery.

The body composition was investigated both for the whole body and for subregions (neck, trunk, arms and legs) according to specific anatomical points of reference. The parameters selected for the purpose of the study were: total fat (Kg), total lean (Kg), and trunk fat (g).

### 2.4. Dietary Intervention

All patients followed a VLCKD (<800 kcal/day) for 45 days, with the following composition in macronutrients and g/kg of ideal BW of proteins (derived from a BMI set at 25 kg/m^2^): carbohydrates < 30 g; olive oil 20 g plus ~15 g of lipids from meals; protein ~90 g (1.2–1.4 g/kg). The amount of protein was within the proposed essential composition of total diet replacements for weight control and was adjusted for patients with overweight or obesity [21]. Patients were given 4–5 meal replacements daily, alongside one portion of low-glycemic index vegetables at lunch and dinner. The number of meal replacements was adjusted according to sex—four per day for women and five for men—to ensure adequate protein intake (see “Supplementary Materials”). Meal replacement consumption was at main meals of 8:00 am, 1:00 pm, and 8:00 pm, mid-morning, and mid-afternoon. Supplements containing vitamins, minerals, and omega-3 fatty acids were provided in accordance with international recommendations [22]. A minimum daily fluid intake of 2 L was recommended. The diets were prepared by New Penta s.r.l. (Cuneo, Italy) following the indications of nutritionists and were delivered in preassembled boxes. Participants received counselling by physicians and nutrition experts at baseline and every two weeks up to the end of treatment; dietary compliance was also assessed by automated measurement of capillary beta-hydroxybutyrate (BOHB) concentrations using a specialized device (GlucoMen Areo 2K, Menarini Diagnostics, Florence, Italy) between 8:00 and 10:00 am in fasting conditions, considering the subject in ketosis if beta-hydroxybutyrate ≥ 0.5 mmol/L [23]. The development of side effects was investigated by the team in charge of the patients every two weeks, focusing on any eventual symptoms, intensity and duration, and need for pharmacological therapies for symptom management. Participants were encouraged to exercise for 30 min at least 3 times weekly, but no formal exercise program was provided.

### 2.5. Data Management and Statistical Methods

Data collection, statistical analysis and graphing were carried out with Microsoft Excel, Statistics-Statsoft software version 12.0 (Stat Soft, Inc., Tulsa, OK, USA) and Prism Graphpad version 10 (Graphpad Software Inc, La Jolla, Boston, MA, US). The data obtained are expressed as mean values ± standard deviation (SD) unless otherwise specified. The Shapiro–Wilk test was used to evaluate the normality of the variables examined. Differences between pre- and post-ketogenic diet were analysed using the dependent samples *t* test. Pearson’s linear regression analysis was used to investigate the correlations between variables. A multivariate linear regression model was used to determine the effect of the variables examined on weight loss. *p* values < 0.05 were considered statistically significant.

## 3. Results

**Figure 1:**
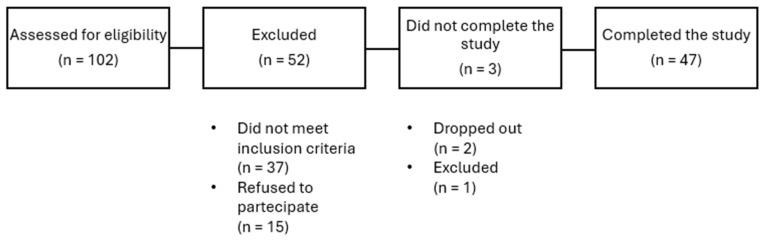
Flow diagram of the study.

A total of 50 subjects affected by overweight or obesity were enrolled. Two patients dropped out for personal reason, and one was excluded for low compliance. The remaining 47 patients completed the study (Figure 1). Among these, 28 were females (60%) and 19 males, with a mean age of 55.5 ± 7.3 years. Concomitant conditions reported were hypertension, dyslipidemia and glucose level alterations. No patient was receiving medications known to affect body weight.

Compliance with the dietary regimen was good among the included patients. Vital signs were stable throughout the study, and no adverse effects of note were reported by any of our patients at follow-up visits.

At baseline, mean BMI was 35.9 ± 4.4 kg/m^2^ and serum OT concentration was 1164 ± 410 pg/mL, with a range from 498 to 2139 pg/mL, and no sex difference was detected (mean OT in females 1147 pg/mL vs. 1193 pg/mL in males). The main characteristics of the study population are reported in Table 1.

**Figure 2:**
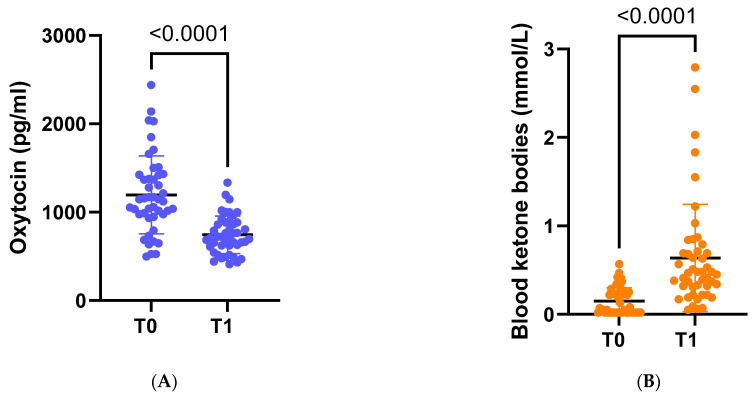
(**A**) Circulating levels of oxytocin at baseline (t0) and at the end of the study (t1). (**B**) Circulating levels of ketone bodies at baseline (t0) and at the end of the study (t1).

Following dietary intervention with VLCKD, anthropometric, biochemical, and body composition parameters were significantly ameliorated. On average, there was a significant weight loss of −8.8 kg, accompanied by a noteworthy decrease in average BMI to 32.7 kg/m^2^ (*p* < 0.001). Furthermore, there was a substantial reduction in OT concentration (*p* < 0.001) (Figure 2A).

After adjustment for age and sex, serum OT levels at baseline (t0) positively correlated with body weight (r = 0.35 *p* < 0.05) and BMI (r = 0.39 *p* < 0.05), but not with waist circumference, nor with waist-to-hip ratio. Regarding metabolic evaluation, baseline OT did not significantly correlate with glucose levels, lipidic profile, or CRP. Regarding body composition, baseline OT levels showed a significant correlation with total fat mass (r = 0.35, *p* < 0.05) and trunk fat (r = 0.33, *p* < 0.05), as shown in Table 2.

**Figure 3:**
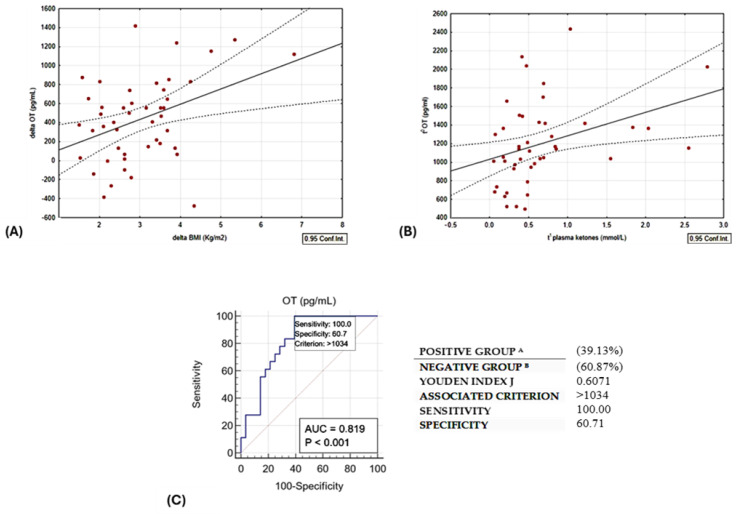
(**A**) Direct linear correlation between change in BMI vs. change in OT, r = 0.3954, *p* = 0.01. (**B**) Direct correlation between OT at t0 and plasma ketones at t1; r = 0.3534, *p* = 0.02. (**C**) ROC curve of circulating t0 OT levels and the occurrence of ketosis (BOHB ≥ 0.5 mmol/L). Group A are patients responders, as they lost more than 10% of initial weight; Group B are reduced responders to the diet intervention.

A linear relationship was observed between delta oxytocin levels and delta BMI, as illustrated in Figure 3A. Baseline OT exhibited a direct correlation with plasma ketone bodies. Notably, this correlation remained statistically significant even after adjusting for age and sex, as depicted in Table 2 and Figure 3B.

Multivariate linear regression analysis shows that OT at baseline independently predicted weight loss (β = 0.36, *p* = 0.02), whereas OT level and plasma ketone concentration measured at the end of the study were not significant predictors (Table 3).

In addition, we plotted the receiver operating characteristic (ROC) curve (Figure 3C) with the Youden index to test the predictive value of baseline circulating OT levels and the occurrence of ketosis at the end of the study. For this purpose, the optimal cut-off of serum OT > 1034 pg/mL could discriminate patients reaching ketosis with a sensitivity and specificity of 100% and 60.7%, respectively (AUC 0.819, *p* < 0.01).

In order to compare the patients who responded differently to the diet intervention, we divided our group in responders, if they lost more than 10% of initial weight, and reduced responders. The two groups did not differ in anthropometric or metabolic characteristics at baseline or at the end of the study, except for baseline oxytocin levels, which were higher in the responder group (1331.55 ± 425.53 vs. 1037.39 ± 422.57 pg/mL, *p* = 0.036); therefore, baseline oxytocin levels may be considered a predictor of weight loss.

## 4. Discussion and Conclusions

Reduced caloric intake combined with increased physical exercise represents the first-line therapeutic approach for obesity management. Establishing healthy nutrition habits and prescribing appropriate dietary strategies that induce weight loss in the short but also in the long term is mandatory in the setting of obesity care, since these can ameliorate many alterations, such as metabolic derangement, fertility issues and sleep disorders [24,25,26,27]. A ketogenic diet (KD) is a peculiar nutritional approach that reshapes the metabolism of the individual, and is characterized by low carbohydrate content and variable protein and fat content. The VLCKD is characterized by a very low content of calories, carbohydrates and fat, and a normal protein content.

OT has recently gained attention for its role in regulating energy balance and promoting pro-social behaviours. In animal models, the administration of oxytocin allows reduced food intake and extended time between meals, leading to reduced caloric intake and ultimately to weight loss [28,29,30]. In the context of obesity, the effects of OT are exerted both centrally, thanks to the widespread distribution of OT-producing neurons and OT receptors (OTR) in areas related to energy balance control, and peripherally [30]. OT is also able to reduce visceral adipose tissue inflammation and stimulate lipolysis and fatty acid beta oxidation [9]. The expression of OTRs in adipocytes is increased in obesity, probably in an attempt to counteract the metabolic and inflammatory alterations of obesity [9].

Circulating OT may therefore play an important role in the regulation of body weight and metabolic homeostasis, since it is implicated in thermoregulation [18] and metabolic processes. In fact, some data suggest that OT influences glucose uptake and metabolism, and that it may stimulate lipolysis and beta-oxidation of fatty acids [15]. Intriguingly, some data in the literature suggest a possible role of OT in promoting adipogenesis in humans [10].

In the literature, we find conflicting data regarding OT levels in obesity, with studies reporting increased [31], normal [32], or decreased values in subjects with obesity [33].

In our study, we observed a positive correlation between OT, weight, and visceral fat mass, consistent with data already collected from previous studies showing higher levels of OT in patients with obesity, and a direct relationship between OT concentrations, BMI and fat mass [13,14,31,34].

Although a change in circulating OT after long-term weight loss is not yet confirmed [13,14], our results show a significant reduction in OT after rapid weight loss (−8.8 kg in 6 weeks) as also reported by other authors [31]. This condition appears to be in contrast with the physiological anorectic role of OT and may be explained by a condition of receptor resistance. If we regard OT as an indicator of energy availability, elevated OT levels would suggest a need to decrease calorie intake and enhance energy expenditure. However, the presence of “OT resistance” can hinder the natural anorectic and weight loss effects of OT, making it necessary for oxytocin to reach higher levels in order to exert its effects. Supporting the theory of OT resistance in obesity, an intriguing study by Brede et al. [35] revealed that, in contrast to individuals with normal weight, individuals with obesity did not experience the expected attenuation of glycemic spikes and insulin excursions after an oral glucose tolerance test following OT administration. Following substantial and rapid weight loss, we observed a significant reduction in OT levels, suggesting that weight loss might have the capacity to restore normal OT–OTR signalling and overcome the hypothesised resistance to the hormone.

Interestingly, we observed that higher baseline oxytocin levels were associated with a more favorable response to the VLCKD intervention. This finding may reflect underlying biological differences among individuals, such as variations in adiposity, metabolic sensitivity, or the functional status of the OT receptor pathway. Individuals with elevated OT levels may exhibit a state of OT resistance. In these subjects, higher OT concentrations could be necessary to activate the OT–OTR signaling pathway, which, once engaged, would exert the expected anorectic effects. The observed reduction in OT levels following weight loss may indicate a decrease in OT resistance.

Understanding the relationship between OT, adipose tissue, and OT signalling in obesity would be essential for better understanding the pathophysiology of a complex disease such as obesity, and hypothesize a role for oxytocin in its treatment.

Another interesting aspect emerging from our study is the correlation between basal levels of OT and the concentration of ketone bodies after 45 days of VLCKD. OT levels could predict the achievement of ketosis as reported by our ROC analysis, and moreover might play a role in enhancing the efficacy of a VLCKD, thanks to OT’s effect on promoting lipolysis and fatty acid beta oxidation [9]. Given the limited sample size, the ROC analysis and the proposed cut-off should be interpreted as exploratory findings and considered a candidate for future validation rather than a tool ready for clinical implementation. Larger, independent studies are required to confirm the robustness and clinical utility of these results. Moreover, although significant associations were observed, the correlational nature of the analyses does not allow any inference regarding causal relationships or causal direction between the variables examined.

Furthermore, our data show that individuals who better responded to the nutritional treatment with a ketogenic diet had higher baseline OT values. Therefore, OT values could be useful for identifying patients that would benefit more from a ketogenic regimen.

Our study presents some limitations, first of all the small sample size and the lack of a control group. Lacking a control group, a key limitation of this study is that the observed efficacy of the intervention in mediating oxytocin levels cannot be definitively attributed to the ketogenic diet itself or to low caloric intake. Because the intervention combined dietary composition and caloric restriction, the independent effects of ketosis versus reduced energy intake cannot be disentangled. In addition, OT is a pulsatile hormone [36], so a single measurement may not be fully representative of each patient’s actual secretory profile. Nevertheless, we standardized the conditions under which the blood samples were taken (e.g., all patients were fasting overnight, the blood samples were taken at the same time for all patients, in the same position, etc.). Moreover, plasma BOHB was measured at baseline and at the end of the study, and some periods without ketosis may have not been detected. However, our primary objective was to highlight changes in OT following weight loss, so ketone measurements were originally intended as a tool to confirm compliance to the diet. Furthermore, the relatively short follow-up period precludes assessment of the persistence of the observed effects over time and limits conclusions regarding the role of oxytocin in long-term weight maintenance. Finally, the exercise level of the patients was not recorded, nor included as a confounding factor in the data analysis.

These things considered, as a strength point, we evaluated the patients with a longitudinal study design.

In conclusion, our data show that OT levels in patients with obesity are significantly reduced after quick weight loss induced by a VLCKD, and that baseline OT levels predict weight loss and correlate with ketone bodies levels achieved with the VLCKD. This evidence may suggest the presence of a state of OT resistance that fast weight loss might restore. Therefore, given the exploratory nature of this study, further research should focus on studying OT in obesity and metabolic derangements in more depth, in order to obtain data to potentially apply in clinical practice.

## Figures and Tables

**Table 1:** Anthropometric, biochemical and body composition parameters of the study population (*n* = 47) at baseline (t0) and after 45 days of VLCKD (t1).

	t0	t1	
Variable	Mean ± DS	Mean ± DS	*p*
Age (years)	55.47 ± 7.33	/	
Weight (Kg)	100.71 ± 13.51	91.49 ± 12.21	<0.01
BMI (kg/m^2^)	35.9 ± 4.4	32.7 ± 3.8	<0.01
WC (cm)	107.84 ± 9.15	101.78 ± 8.45	<0.01
HC (cm)	124.08 ± 10.46	118.55 ± 10.54	<0.01
Hb1Ac (%)	5.72 ± 0.58	5.46 ± 0.42	0.02
Glucose (mg/dL)	105.91 ± 19.07	95.80 ± 13.09	<0.01
Insulin (uU/mL)	19.50 ± 11.89	7.96 ± 3.95	<0.001
Total Cholesterol (mg/dL)	220.85 ± 39.57	183.66 ± 36.38	<0.001
cLDL (mg/dL)	137.25 ± 33.18	112.34 ± 30.70	<0.001
HDL (mg/dL)	53.91 ± 17.06	51.80 ± 13.14	n.s.
TG (mg/dL)	141.66 ± 86.18	98.16 ± 39.12	<0.01
CRP (mcg/L)	5545.41 ± 6754.27	4218.54 ± 4557.32	n.s.
TRUNK-Fat (Kg)	18.12 ± 4.17	15.74 ± 4.	<0.01
Fat Tot (Kg)	36.91 ± 8.90	32.04 ± 8.92	<0.01
Lean Tot (Kg)	60.79 ± 9.84	58.64 ± 9.94	n.s.

Abbreviations: WC, waist circumference; HC, hip circumference; BMI, body mass index; Hb1Ac, glycated hemoglobin; LDL, low-density lipoprotein; HDL, high-density lipoprotein; TG, triglycerides; CRP, C reactive protein; OT, oxytocin; n.s.: non-significant.

**Table 2:** Linear correlations between baseline OT levels and anthropometric data, body composition parameters and blood ketone levels, after adjustment for age and sex. Only significant correlations are reported.

	Pearson’s Correlation		
	*R*	95% CI	*p*
OT t0 (pg/mL)			
BMI (kg/m^2^)—t0	0.39	−0.012–0.55	0.01
Total fat (Kg)—t0	0.35	0.08–0.62	0.04
Total mass (kg)—t0	0.39	0.03–0.58	0.03
Trunk fat (g)—t0	0.33	0.19–0.69	0.05
Blood ketones (mmol/L)—t1	0.36	0.07–0.58	0.02

Abbreviations: OT, oxytocin; BMI, body mass index.

**Table 3:** Multiple linear regression—dependent variable Δ BMI t0–t1.

	R = 0.510 R^2^ = 0.260 Adjusted R^2^ = 0.165					
	b *	Std. Err. of b *	b	Std. Err. of b	t(39)	*p*-Value
Intercept			1.36	1.23	1.10	0.27
Sex	0.06	0.14	0.13	0.30	0.42	0.68
Years (age)	0.05	0.14	0.01	0.02	0.33	0.74
OT pg/mL t0	0.36	0.15	<0.01	<0.01	2.39	0.02
OT pg/mL t1	−0.04	0.14	<−0.01	<0.01	−0.25	0.80
Plasma ketones mmol/L t1	0.24	0.15	0.41	0.25	1.63	0.11

Abbreviations: OT, oxytocin.b *: the intercept of the regression line.

## Data Availability

Data is available from the last author upon a reasonable request.

## Supplementary Materials

The following supporting information can be downloaded at: https://www.mdpi.com/article/10.3390/nu18030485/s1, Table S1: Dietary recall.
