# Supplementary material for: Oxytocin, Weight Loss and Ketosis in Response to a Very-Low-Calorie Ketogenic Diet: An Exploratory Study
Source: Nutrients. 2026 Feb 1;18(3):485. doi: 10.3390/nu18030485 (PMC12899579; doi:10.3390/nu18030485)
Supplement: Supplementary file 1 [file nutrients-18-00485-s001.zip › nutrients-4054399-supplementary.pdf]

Supplementary Material

Table S1: Dietary recall

	Male patients	Female patients
**Breakfast**	1 ketogenic meal (e.g. chocolate drink or cappuccino drink)	1 ketogenic meal (e.g. chocolate drink or cappuccino drink)
**Mid-morning snack**	1 ketogenic snack (e.g. biscuits, one sweet snack, one salty snack)<br>+<br>Supplements with 200 ml of water	Supplements with 200 ml of water
**Lunch**	1 ketogenic meal (e.g. ketogenic pasta, or risotto, or bread, or omelette or meatballs)<br>+<br>One portion of low-glycaemic index vegetables with one tablespoon of olive oil	1 ketogenic meal (e.g. ketogenic pasta, or risotto, or bread, or omelette or meatballs)<br>+<br>One portion of low-glycaemic index vegetables with one tablespoon of olive oil
**Mid-afternoon snack**	1 ketogenic snack (e.g. biscuits, one sweet snack, one salty snack)	1 ketogenic snack (e.g. biscuits, one sweet snack, one salty snack)
**Dinner**	1 ketogenic meal (e.g. ketogenic pasta, or risotto, or bread, or omelette or meatballs)<br>+<br>One portion of low-glycaemic index vegetables with one tablespoon of olive oil	1 ketogenic meal (e.g. ketogenic pasta, or risotto, or bread, or omelette or meatballs)<br>+<br>One portion of low-glycaemic index vegetables with one tablespoon of olive oil
